# Sensitivity and concurrent validity of the Japanese version of the Euthymia scale: a clinimetric analysis

**DOI:** 10.1186/s12888-021-03494-7

**Published:** 2021-10-04

**Authors:** Natsu Sasaki, Danilo Carrozzino, Daisuke Nishi

**Affiliations:** 1 Department of Mental Health, Graduate School of Medicine, The University of Tokyo, 7-3-1, Hongo, Bunkyo-ku, Tokyo, 113-0033 Japan; 2 Department of Psychology “Renzo Canestrari”, Alma Mater Studiorum, University of Bologna, Bologna, Italy

**Keywords:** Clinimetrics, Depression, Euthymia, Psychological distress, Sensitivity, Validity

## Abstract

**Background:**

Euthymia is characterized by the lack of mood disorders, the presence of positive affects, psychological flexibility and well-being, a unifying outlook on life, and resistance to stress. The Euthymia Scale (ES) is a 10-item self-rating clinimetric index assessing euthymia.

**Objectives:**

The present study was conducted to examine the clinimetric sensitivity and concurrent validity of the Japanese version of the Euthymia Scale (ES-J).

**Methods:**

A cross-sectional online survey was conducted. The Mini-International Neuropsychiatric Interview was used to determine the presence of past or current major depressive episodes (MDE). The clinimetric sensitivity was evaluated using the Analysis of Variance (ANOVA). Pearson’s correlation coefficients were performed to examine the concurrent validity of the ES-J.

**Results:**

A total of 1030 eligible participants completed the survey. The ES-J differentiated healthy subjects from complete remission (i.e., those with a past history of MDE without current MDE) (*p* < 0.001), from those with past or current history of MDE (*p* < 0.001), subjects with current MDE from those with sub-threshold symptoms of depression (*p* < 0.001), and healthy participants from subjects with moderate to severe symptoms of psychological distress (*p* < 0.001). The associations between the ES-J and measures of psychological well-being, resilience, life satisfaction, and social support were significantly positive (0.353 < r < 0.666, *p* < 0.001). A negative relationship between the ES-J and measures of psychological distress was also found (r = − 0.595, *p* < 0.001).

**Conclusions:**

The findings of the present study indicated that the ES-J is a valid and highly sensitive clinimetric index, which can be used as a screening measure in the clinical process of assessment of recovery, particularly when symptoms are expected to be mild and/or when dealing with subclinical symptoms of psychological distress and depression. The findings of this study also support the use of the ES-J to detect vulnerability to depression and to identify subjects at higher risk of relapse.

## Highlights

• The ES-J was a highly sensitive clinimetric index.

• The ES-J distinguished healthy subjects from those with past or current major depression.

• The ES-J was highly sensitive to sub-threshold symptoms of depression.

• The ES-J was a valid screening measure.

• The ES-J can be used to detect a kind of vulnerability and risk of relapse.

## Introduction

The evaluation of current depression and the assessment of the risk of relapse require highly sensitive rating scales that can be used in a variety of clinical and research settings. It should be noted, however, that most of the existing rating scales that have been developed according to the psychometric assumption of homogeneity of components were found to display not high sensitivity in the clinical process of assessment of depression [1]. A sensitive assessment instrument is thus expected to be validated.

The concept of “sensitivity”, which was originally introduced by Kellner [2], refers to the ability of the rating scale to (1) detect clinically relevant changes in clinical (i.e., drug and psychotherapy) trials discriminating between active treatment and placebo, (2) to differentiate the severity of symptoms (e.g., certain symptoms may be more troublesome or incapacitating than others), (3) to detect residual and subclinical symptoms, (4) to differentiate patients from controls (i.e., healthy subjects), and (5) to discriminate between different groups of patients suffering from the same illness (e.g., depressed inpatients versus depressed outpatients). The assessment of sensitivity has been considered a central issue in clinimetrics [3–7].

Alvan R. Feinstein [8, 9] coined the term “clinimetrics” to introduce an innovative approach, which has been defined as the science of clinical measurements [10]. Clinimetrics has a set of criteria, which are used not only to develop new clinimetric indices but also to establish the validity, sensitivity, and clinical utility of existing rating scales [11]. Such a set of principles has been recently refined with the introduction of Clinimetric Patient-Reported Outcome Measures (CLIPROM) criteria [12], which represent a step forward to the assessment of clinical properties of rating scales. Following clinimetric criteria, which significantly differ from those traditionally used in the psychometric model [11], Fava and Bech [13] developed a clinimetric index, the Euthymia Scale (ES), a 10-item self-rating scale assessing euthymia. The concept of euthymia, as originally introduced by Fava and Bech (2016) and recently refined by Fava and Guidi [14], refers to a transdiagnostic construct characterized not only by the lack of mood disorders but, most importantly, by the presence of positive affects, psychological flexibility and well-being, a unifying outlook on life, and resistance to stress (i.e., resilience and tolerance to anxiety or frustration).

Carrozzino et al. (2019) recently conducted a study showing that the Italian version of the ES was found to entail the clinimetric properties of the construct as well as concurrent and incremental validities [7]. No data are, however, available on the ability of the ES to discriminate between different groups of subjects; therefore, a further study was needed to evaluate the sensitivity of the ES.

Using a large sample of participants recruited from the general population of Japan, including subjects with a past or current history of major depression, the present study was conducted to primarily examine the clinimetric sensitivity of the Japanese version of the ES (ES-J). The concurrent validity of the ES-J was also tested. As to the internal reliability or consistency, CLIPROM criteria were used [12], implying that this measurement property was not evaluated, as the items of the ES-J are not assumed to be inter-correlated.

## Methods

### Study design

The study utilized a cross-sectional design and collected the data using an online survey. The Ethical Committee of the University of Tokyo (Graduate School of Medicine and Faculty of Medicine), Japan (Institutional review board No. 2019361NI), approved the study protocol.

### Participants

The participants were drawn from registered members of an online survey site, Macromill, Inc. [15]. The company had access to over 2,300,000 potential participants representing all prefectures in Japan and recruited participants based on their demographic attributes to obtain a relatively representative sample. Of the available respondents, a stratified random sample of 1030 participants completed a web-based questionnaire in order of arrival to the form. Participants were sampled from two strata equally (50% vs. 50%) according to their history of major depressive episodes (MDE), as evaluated by the Mini International Neuropsychiatric Interview (MINI): total scores≧5 or 0≦total scores≦4. Participants had to meet the following criteria to be included in the study: (a) living in Japan and (b) being 20 years of age or older. There were no exclusion criteria. Based on these criteria, the Internet survey company recruited monitors from their potential pool of participants until the targeted number was reached. Participating monitors were awarded approximately 100 tokens (equivalent to 100 Japanese yen) as a reward. Informed consent was obtained from all participants via instructions on the survey. The instructions assured the protection of personal information and explained that any identifying information would be removed from the data.

### Assessment instruments

#### Euthymia scale (ES)

The Japanese version of the ES, the ES-J, was obtained according to the procedure specified in the International Society of Pharmacoeconomics and Outcomes Research (ISPOR) task force guidelines [16]. First, we obtained permission from the original developer of the Euthymia Scale (Professor Giovanni A. Fava) to translate the measure into Japanese [13]. Forward-translation was conducted independently by two Japanese authors (Natsu Sasaki and Daisuke Nishi) and was followed by reconciliation, back-translation, back-translation review, harmonization, and cognitive debriefing. The Japanese version of the 5-item World Health Organization Well-Being Index (WHO-5) was referred to in the latter half of the ES (No.6 to No.10) in forward-translation [17]. The back-translation was conducted by an expert in Japanese and English affiliated with the University of Tokyo who did not know the purpose of the present study. The original developer checked the back-translated measure and confirmed it at the back-translation review. Cognitive debriefing sessions were conducted with six general Japanese people who were recruited using snowball sampling and included graduate students who specialized in mental health, a psychiatrist, and office workers, whose ages ranged from the 20s to 50s. They were asked to complete the harmonized measure and were interviewed about the relevance, comprehensiveness, and comprehensibility of the items. The cognitive debriefing process did not lead to any change in wording. The authors confirmed the cognitive equivalence of the translated ES-J (the scale can be available by contacting the corresponding author).

The original version of the ES-J rating scale is a 10-item self-reported questionnaire. Each item of the ES-J is scored dichotomously as *False* (0) or *True* (1), resulting in an overall score ranging from 0 to 10, with higher scores indicating a better euthymic state.

#### Mini-international neuropsychiatric interview (MINI)

The MINI is a widely used clinician-rated scale (i.e., structured interview) for the assessment of axis I psychiatric disorders according to DSM-IV diagnostic criteria [18–21]. Diagnoses are based on dimensional scores (Yes or No) obtained from nine items (e.g., Were you [ever] depressed or down, or felt sad, empty or hopeless most of the day, nearly every day [in lifetime/for the past two weeks]?). Authors measured history of MDE in lifetime and current MDE in recent two weeks, using 9 items respectively. History of MDE in lifetime was categorized as Yes (score 5–9) or None (score 0–4). According to the Diagnostic and Statistical Manual of mental disorders (DSM-5), current MDE (endorsed 5–9 items), sub-threshold depression (endorsed 1–4 items), and none (endorsed 0 items) were used as criteria among the total of 9 items [18].

#### Psychological distress

Psychological distress was evaluated using the Japanese version of the K6 [22, 23]. The K6 is a widely used self-rating scale assessing nonspecific distress during the past 30 days. Each item of the K6 is scored on a Likert scale ranging from *never* (0) to *all of the time* (4). The total score of the K6 ranges from 0 to 24, with higher scores indicating more severe psychological distress. A score of more than 13 on the K6 was used to detect severe symptoms of psychological distress. A K6 score of more than 5 was indicative of moderate symptoms of mental distress [24].

The validity of the Japanese version of the K6 was found to be satisfactory [22, 25].

#### Psychological well-being

Psychological well-being was evaluated using the 42-item version of the Psychological Well-being Scales (PWBS) developed by Carol D. Ryff. The PWBS originally consisted of six subscales, each including seven items, assessing the following six factors: 1) autonomy; 2) environmental mastery; 3) personal growth; 4) positive relations with others; 5) purpose in life; and 6) self-acceptance [26, 27]. Response categories for these items are on a seven-point Likert scale ranging from *Strongly disagree* (1) to *Strongly agree* (7). The scores of some items were reversed as recommended in Ryff’s original PWBS [26, 27]. The average scores were calculated for six subscales, with higher mean scores indicating greater psychological well-being. The validity of the Japanese version of PWBS has been recently tested [28].

#### Resilience

Resilience was evaluated using the Tachikawa Resilience Scale (TRS) [29, 30]. TRS is a 10-item self-administered scale. All items are rated on a 7-point Likert scale, ranging from *strongly disagree* (1) to *strongly agree* (7). The total scores ranged from 10 to 70, with higher scores reflecting higher resilience.

The original TRS was in the Japanese language, and several items reflected Japanese culture-bound cognitions. For example, items such as, “I accept things as they are when there are no alternatives” and “I try not to worry about what is beyond my capabilities” can be regarded as culturally appropriate for Japanese individuals because these items reflect the idea of Morita therapy, which guides patients to accept anxiety as it is [30]. The validity of the TRS was acceptable [29].

#### Life satisfaction

The Satisfaction With Life Scale (SWLS), developed by Diener [31], was used to measure life satisfaction. The SWLS is a 5-item broad-band instrument measuring life satisfaction. Examples of items are, “In most ways, my life is close to my ideal,” and “If I could live my life over, I would change almost nothing.” The SWLS uses a 7-point Likert scale, ranging from *strongly disagree* (1) to *strongly agree* (7), yielding a total score ranging from 5 (low life satisfaction) to 35 (high life satisfaction). The validity of SWLS was acceptable [32, 33].

#### Social support

Social support was assessed using the Japanese short (7-item) version of the self-rated Multidimensional Scale of Perceived Social Support (MSPSS) [34, 35]. It assesses perceived support from each of three sources: family (2 items), friends (3 items), and a significant other (2 items). The items are measured on a 7-point Likert scale ranging from *very strongly disagree* (1) to *very strongly agree* (7), with higher scores suggesting greater levels of perceived social support. The mean score of 7 items was used as a total score.

#### Demographic variables

A questionnaire was administered to assess the following demographic variables: gender (male or female), age, marital status (married, divorced/widowed or single), having a child, household income, and education status (Junior high school, high school, college, undergraduate school, upper than graduate school).

### Statistical analyses

Statistical significance was defined as *p* < 0.05. All the statistical analyses were performed using SPSS 26.0, Japanese version (IBM Inc., Chicago, IL).

Pearson’s correlation coefficients (*r*s) were calculated to examine the concurrent validity of the ES-J. Positive and moderate to high correlations were expected with rating scales measuring psychological well-being, resilience, life satisfaction, and social support. Negative and moderate to high correlations were expected with measures of psychological distress.

Analysis of variance (ANOVA) was conducted to examine the clinimetric sensitivity of the ES-J and test whether this rating scale sensitively distinguishes moderate from severe symptoms of psychological distress and discriminates between patients (i.e., those with past or current history of MDE or with sub-threshold symptoms of depression) and healthy subjects.

The Jonckheere’s test was conducted to examine the trend of ES-J among the groups.

## Results

### Characteristics of participants

A total of 1030 subjects completed the online survey. The proportion of males was 47.4%, and the mean age was 46.8 years old. Demographic characteristics and variables categorized by the presence of a history of MDE are shown in Table 1. The mean scores on the ES-J and other rating scales are also presented in Table 1. Subjects without a history of MDE scored significantly (*p* < 0.001) higher on the ES-J (mean = 7.35) compared to those with a history of MDE (mean = 4.57).

**Table 1:** Participants’ characteristics of online survey in Japan screened and mean scores of euthymia scale, psychological well-being (PWB) scale, resilience, life satisfaction, and social support by past major depressive episodes (MDE) measured by self-reported the Mini International Neuropsychiatric Interview (M.I.N.I.) (*N* = 1030)

	History of MDE		*p*-value†
	None (*n* = 515)	Yes (*n* = 515)	
	N (%)	N (%)	
Sex male	240 (46.6)	248 (48.2)	0.618
Age mean (SD) [min - max]	46.8 (13.5) [20–88]	46.8 (13.5) [20–88]	< 0.001**
20–24 years old	16 (3.1)	18 (3.5)	
25–29	32 (6.2)	50 (9.7)	
30–34	46 (8.9)	62 (12.0)	
35–39	44 (8.5)	60 (11.7)	
40–44	44 (8.5)	61 (11.8)	
45–49	80 (15.5)	90 (17.5)	
50–54	80 (15.5)	66 (12.8)	
55–59	49 (9.5)	54 (10.5)	
> 60	124 (24.1)	54 (10.5)	
Married	355 (68.9)	293 (56.9)	< 0.001**
Having a child/ children	325 (63.1)	270 (52.4)	0.001**
Household income			0.833
< 2 million yen	31 (6.0)	36 (7.0)	
2–4	89 (17.3)	83 (16.1)	
4–6	116 (22.5)	108 (21.0)	
6–8	78 (15.1)	75 (14.6)	
8–10	40 (7.8)	53 (10.3)	
10<	42 (8.2)	40 (7.8)	
unknown	119 (23.1)	120 (23.3)	
Education status			0.012*
Junior high school	15 (2.9)	11 (2.1)	
High school	173 (33.6)	132 (25.6)	
College	126 (24.5)	116 (22.5)	
Undergraduate school	187 (36.3)	232 (45.0)	
Upper than graduate school	14 (2.7)	23	
Unknown	-(−)	1 (0.2)	
Scale [range]	Mean (SD)	Mean (SD)	
Euthymia scale [0–10]	7.35 (2.54)	4.57 (2.86)	< 0.001**
PWB			
Autonomy [7–49]	29.86 (5.46)	27.6 (6.45)	< 0.001**
Environmental mastery [7–49]	30.93 (5.01)	26.0 (5.88)	< 0.001**
Personal growth [7–49]	31.43 (5.77)	27.9 (6.88)	< 0.001**
Positive relationships with others [7–49]	31.43 (5.66)	27.7 (6.97)	< 0.001**
Purpose in life [7–49]	30.02 (4.28)	28.0 (4.94)	< 0.001**
Self-acceptance [7–49]	30.08 (5.70)	24.9 (7.09)	< 0.001**
Resilience [10–70]	46.1 (9.22)	38.9 (10.5)	< 0.001**
Life satisfaction [5–35]	20.6 (6.05)	16.0 (6.85)	< 0.001**
Social support [1–7]	5.01 (1.27)	4.26 (1.53)	< 0.001**

† Chi-test, variance analysis, or regression analysis was conducted to detect the difference between each group

*MDE* major depressive episode. *SD* standard deviation

** *p* < 0.01, * < 0.05

### Concurrent validity

Table 2 shows correlation analyses. The ES-J was found to be positively and significantly correlated (0.353 < r < 0.666, *p* < 0.001) with measures of positive mental health (i.e., dimensions of psychological well-being, resilience, life satisfaction, and social support). A negative and statistically significant relationship (r = − 0.595, *p* < 0.001) between the ES-J and measures of psychological distress (K6) was found.

**Table 2:** Pearson’s correlation coefficients between euthymia scale and other variables (*N* = 1030)

Variables	2	3	4	5	6	7	8	9	10	11	12
1. Euthymia scale	.656**	.428**	.631**	.557**	.512**	.353**	.614**	.666**	.544**	.476**	−.595**
2. Psychological well-being (all)		.658**	.866**	.859**	.816**	.670**	.884**	.638**	.657**	.623**	−.621**
3. Autonomy			.563**	.450**	.322**	.270**	.519**	.457**	.302**	.171**	−.348**
4. Environmental mastery				.659**	.665**	.470**	.749**	.591**	.578**	.518**	−.647**
5. Personal growth					.666**	.599**	.700**	.519**	.508**	.525**	−.502**
6. Positive relationships with others						.492**	.695**	.508**	.587**	.729**	−.492**
7. Purpose in life							.490**	.291**	.401**	.420**	−.378**
8. Self-acceptance								.632**	.717**	.576**	−.576**
9. Resilience									.568**	.480**	−.488**
10. Life satisfaction										.626**	−.449**
11. Social support											−.426**
12. Psychological distress											

** *p* < 0.01

### Clinimetric sensitivity

Mean scores of the ES-J individual items and total score stratified according to the past or current history of MDE are reported in Table 3. All but one item (i.e., item no. 4) of the ES-J significantly discriminated healthy subjects from those with a past or current history of MDE. The same trend was observed when using the total score: the ES-J differentiated between healthy participants (Group 0: past -, current -) and those in complete remission (Group1: past +, current -) (*p* < 0.001), discriminated healthy subjects from those with past or current history of MDE (*p* < 0.001), and sensitively differentiated between subjects with sub-threshold symptoms of depression and individuals with current MDE (*p* < 0.001).

**Table 3:** The mean score and standard deviation of each item of the Japanese version of the Euthymia scale stratified the categories by the history of MDE and current MDE (*N* = 1030)

	Mean (SD)						Test for difference	
	Group	0	1	2	3	4		
Item description	Total	Past (−)† Current (−)‡	Past (+)† Current (−)‡	Past (−)† Current (±) ‡	Past (+)† Current (±) ‡	Past (+)† Current (+)‡		
	*N* = 1030	*N* = 500	*N* = 311	*N* = 15	*N* = 29	*N* = 175	*p*-value§	F value
1. If I become sad, anxious or angry it is for a short time	0.63 (0.5)	0.79 (0.4)	0.52 (0.5)	0.67 (0.5)	0.48 (0.5)	0.39 (0.5)	< 0.001	33.56
2. I do not keep thinking about negative experiences	0.51 (0.5)	0.68 (0.5)	0.39 (0.5)	0.53 (0.5)	0.34 (0.5)	0.26 (0.4)	< 0.001	34.30
3. I am able to adapt to changing situations	0.63 (0.5)	0.78 (0.4)	0.55 (0.5)	0.80 (0.4)	0.48 (0.5)	0.34 (0.5)	< 0.001	34.50
4. I try to be consistent in my attitudes and behaviors	0.77 (0.4)	0.78 (0.4)	0.78 (0.4)	0.73 (0.5)	0.79 (0.4)	0.73 (0.4)	0.722	0.52
5. Most of the time I can handle stress	0.55 (0.5)	0.73 (0.4)	0.46 (0.5)	0.53 (0.5)	0.52 (0.5)	0.19 (0.4)	< 0.001	51.28
6. I generally feel cheerful and in good spirits	0.64 (0.5)	0.81 (0.4)	0.58 (0.5)	0.73 (0.5)	0.52 (0.5)	0.28 (0.5)	< 0.001	49.53
7. I generally feel calm and relaxed	0.71 (0.5)	0.85 (0.4)	0.66 (0.5)	0.87 (0.4)	0.55 (0.5)	0.39 (0.5)	< 0.001	42.50
8. I generally feel active and vigorous	0.51 (0.5)	0.65 (0.5)	0.47 (0.5)	0.47 (0.5)	0.41 (0.5)	0.17 (0.4)	< 0.001	35.47
9. My daily life is filled with things that interest me	0.54 (0.5)	0.64 (0.5)	0.51 (0.5)	0.53 (0.5)	0.34 (0.5)	0.32 (0.5)	< 0.001	15.80
10. I wake up feeling fresh and rested	0.48 (0.5)	0.66 (0.5)	0.39 (0.5)	0.47 (0.5)	0.28 (0.5)	0.18 (0.4)	< 0.001	42.97
Total scores	5.96 (3.04)	7.38 (2.53)	5.29 (2.79)	6.33 (2.64)	4.72 (2.85)	3.26 (2.53)	< 0.001 ※	89.90

§ *p*-value for ANOVA

※ < 0.001** Group0 x Group1 < 0.001** Group0 x Group3 < 0.001** Group0 x Group4 < 0.001** Group1 x Group4 < 0.001** Group2 x Group4

Marginally *p* = 0.054 Group3 x Group4

†Past (+): total scores≧5, Past (−): 0≦total scores≦4, measured by The Mini International Neuropsychiatric Interview (M.I.N.I.) questionnaire for lifetime episode

‡Current(+): total scores≧5, Current (±): 1≦total score≦4, Current (−): score = 0 by The Mini International Neuropsychiatric Interview (M.I.N.I.) questionnaire for current 2 weeks episode

*ES* euthymia scale. *SD* standard deviation

Table 4 shows the results of the same analysis stratified by history of MDE and psychological distress (K6 scores). All ES-J items were found to discriminate sensitively between severe and moderate symptoms of psychological distress. Among subjects with a history of MDE, the ES-J sensitively discriminated participants with moderate symptoms of psychological distress from those with severe psychological distress (*p* < 0.001). The ES-J also differentiated healthy respondents (i.e., those with no history of MDE and without psychological distress) from subjects with a history of MDE and moderate to severe symptoms of psychological distress (*p* < 0.001).

**Table 4:** The mean score and standard deviation of each item of the Japanese version of the Euthymia scale stratified the categories by the history of MDE and psychological distress (*N* = 1030)

	Mean (SD)							Test for difference	
	Group	0	1	2	3	4	5		
Item description	Total	Past (−) † K6 (0–4)	Past (+) † K6 (0–4)	Past (−) † K6 (5–12)	Past (+) † K6 (5–12)	Past (−) † K6 (≧13)	Past (+) † K6 (≧13)		
	*N* = 1030	*N* = 327	*N* = 90	*N* = 165	*N* = 222	*N* = 23	*N* = 203	*p*-value§	F value
1. If I become sad, anxious or angry it is for a short time	0.63 (0.5)	0.83 (0.4)	0.71 (0.5)	0.73 (0.4)	0.50 (0.5)	0.61 (0.5)	0.34 (0.5)	< 0.001	36.83
2. I do not keep thinking about negative experiences	0.51 (0.5)	0.76 (0.4)	0.53 (0.5)	0.56 (0.5)	0.39 (0.5)	0.35 (0.5)	0.20 (0.4)	< 0.001	41.14
3. I am able to adapt to changing situations	0.63 (0.5)	0.83 (0.4)	0.71 (0.5)	0.71 (0.5)	0.50 (0.5)	0.52 (0.5)	0.34 (0.5)	< 0.001	36.37
4. I try to be consistent in my attitudes and behaviors	0.77 (0.4)	0.80 (0.4)	0.87 (0.3)	0.72 (0.4)	0.79 (0.4)	0.78 (0.4)	0.69 (0.5)	0.006	3.31
5. Most of the time I can handle stress	0.55 (0.5)	0.82 (0.4)	0.61 (0.5)	0.59 (0.5)	0.42 (0.5)	0.43 (0.5)	0.20 (0.4)	< 0.001	53.44
6. I generally feel cheerful and in good spirits	0.64 (0.5)	0.89 (0.3)	0.73 (0.4)	0.70 (0.5)	0.55 (0.5)	0.39 (0.5)	0.29 (0.5)	< 0.001	55.11
7. I generally feel calm and relaxed	0.71 (0.5)	0.92 (0.3)	0.81 (0.4)	0.77 (0.4)	0.60 (0.5)	0.48 (0.5)	0.41 (0.5)	< 0.001	45.39
8. I generally feel active and vigorous	0.51 (0.5)	0.75 (0.4)	0.68 (0.5)	0.49 (0.5)	0.39 (0.5)	0.35 (0.5)	0.20 (0.4)	< 0.001	43.51
9. My daily life is filled with things that interest me	0.54 (0.5)	0.71 (0.5)	0.64 (0.5)	0.53 (0.5)	0.45 (0.5)	0.35 (0.5)	0.33 (0.5)	< 0.001	19.59
10. I wake up feeling fresh and rested	0.48 (0.5)	0.77 (0.4)	0.59 (0.5)	0.49 (0.5)	0.33 (0.5)	0.30 (0.5)	0.16 (0.4)	< 0.001	55.32
Total scores	5.96 (3.04)	8.08 (2.29)	6.89 (2.52)	6.30 (2.41)	4.93 (2.59)	4.57 (2.45)	3.14 (2.48)	※ < 0.001	116.22

§ *P*-value for ANOVA

※Statistically significant differences were found

< 0.001** Group0 x Group2 < 0.001** Group0 x Group3 < 0.001** Group0 x Group4 < 0.001** Group0 x Group5

< 0.001** Group1 x Group3 < 0.001** Group1 x Group5 < 0.001** Group2 x Group3 < 0.001** Group2 x Group5

< 0.001** Group3 x Group5

*P* = 0.001 Group0 x Group1 *P* = 0.001 Group1 x Group4

*P* = 0.021 Group2 x Group4

There was no significant difference between Group1–2, 3–4 and 4–5

†Past (+): total scores≧5, Past (−): 0≦total scores≦4, measured by The Mini International Neuropsychiatric Interview (M.I.N.I.) questionnaire for lifetime episode

*ES* euthymia scale. *SD* standard deviation

The Jonckheere trend test revealed a significant trend (*p* < 0.001): a healthy population without history scored higher on ES-J, and a severely distressed population with history scored lower on ES-J.

## Discussion

This study found that the ES-J discriminated healthy subjects from participants with past or current MDE, or with mild/severe psychological distress regardless of history of MDE. The ES-J also discriminated subjects with current MDE from those with sub-threshold symptoms of depression. ES-J correlated positively with measures of positive mental health and negatively with psychological distress. More specifically, the findings of the ANOVA indicated that the ES-J is a highly sensitive clinimetric index, as it discriminated between healthy participants and respondents with moderate to severe symptoms of psychological distress, differentiated healthy subjects from those with a past or current history of MDE, and, most importantly, distinguished subjects with current MDE from those with sub-threshold symptoms of depression. The ability to detect subclinical or residual symptoms after remission is particularly important in the clinical process of assessment of recovery, as such symptoms hinder lasting recovery and are among the strongest risk factors for relapse [36–38]. Thus, the ES-J seems to provide a comprehensive assessment of recovery, particularly when symptoms are expected to be mild and/or when dealing with subclinical symptoms. The findings of the present study also support the use of the ES-J as a highly sensitive screening measure in detecting vulnerability to depression and to identify subjects at higher risk of relapse. Future studies are, however, needed to confirm the predictive validity of the ES-J and to examine whether subjects with lower ES-J scores will relapse or develop affective disorders. This study did not show the significant difference for ES-J scores between healthy subjects and those with sub-threshold symptoms of depression, and between subjects with a history of MDE with no psychological distress and those with mild distress without history of MDE. A total score of 10 points may not be sufficient to recognize very small differences in conditions, and more detailed examination, such as the pattern of scores, may be necessary. One item (item no. 4) of the ES-J showed relatively small differences among the groups compared to other items. The item no.4 of “I try to be consistent in my attitudes and behaviors” reflected the psychological ability to maintain balance among important life domains, and to display consistency in one’s behavior and deeply held values [13], however, Japanese population might have assumed that they would change their opinion or responses depending on the situation and the person they were talking to. Cultural amendment may, therefore, be considered in the future investigations.

The ES-J was also found to have excellent concurrent validity, as it correlated positively with measures of positive mental health (i.e., psychological well-being, resilience, life satisfaction, and social support scales) and negatively with psychological distress. These findings are in the expected direction, consistent with the original concept of euthymia originally introduced by Fava and Bech (2016). They proposed a construct beyond the traditional concept of euthymia [13, 14]. As Fava and Guidi (2020) noted, “In the psychiatric literature, the term euthymia essentially connotes the lack of significant distress” (p. 42). Such a traditional concept of euthymia clashed with the clinical reality, where a state of euthymia implies not only the absence of psychological distress but also the presence of positive affects and psychological well-being [13, 14]. Fava and Guidi (2020) also noted, “When a patient, in the longitudinal course of mood disturbances, no longer meets the threshold for a disorder such as depression or mania, as assessed by diagnostic criteria or by cut-off points on rating scales, he/she is often labeled as euthymic. However, considerable fluctuations in psychological distress were recorded in studies with longitudinal designs, suggesting that the illness is still active in those latter periods, even though its intensity may vary” (p. 42). The euthymia scale was specifically developed to capture such fluctuations [13, 14]. The findings of the present study indeed revealed that the ES-J is highly sensitive to such fluctuations and able to differentiate the severity of symptoms of psychological distress. The evidence that the ES-J was found to be correlated to the K6 is also in line with recent research studies [39–41] that showed a statistically significant and negative relationship between dimensions of euthymia (e.g., psychological flexibility and resilience) and components of psychological distress (e.g., mental pain and neuroticism).

### Limitations

This study has some limitations. First, the cross-sectional design of the present study limited the evaluation of test-retest reliability and conclusions regarding causality. Future studies should use a longitudinal design to assess the predictive and incremental validity of the ES-J.

Second, we did not use item response theory (IRT) models (i.e., Rasch and Mokken analyses) to evaluate the scalability or dimensionality of the ES-J. Such a clinimetric property has been considered an important issue in clinimetrics. It refers to the evaluation of the extent to which the total score of the rating scale is a statistically sufficient and clinically valid measure of the clinical condition under examination [42].

Third, the ES-J was administered only to participants from the general population, thus limiting the generalizability of the findings. The generalizability was also limited because participants were recruited through an online survey without a clinical assessment of MDE. Online recruitment may cause selection bias, such as high IT and health literacy and device ownership, further limiting the generalizability of study findings. All outcomes in the present study were indeed evaluated using only self-reported questionnaires, thus limiting the clinical validity of study findings.

### Implications for future studies

For further understandings of the clinical characteristics of the ES-J, a follow-up study is needed to examine its stability and predictive validity, particularly the power of this clinimetric index to predict the relapse of depression. Besides, cultural differences in ES may need to be considered. Future studies investigating the cross-cultural validity of the ES-J are, therefore, needed, particularly to evaluate its clinimetric transferability or applicability to different cultures. Investigating whether the specific score patterns reflect on psychiatric conditions or using other response options (i.e., multiple) may also lead to innovative findings. The pursuit of euthymia has been considered one of the most important trans-diagnostic strategies to be employed in psychotherapy research and practice [43]. Thus, future studies evaluating the clinical utility of the ES-J in psychotherapy trials are highly encouraged. The relationship between euthymia and other psychosomatic diseases instead of depression should be also tested, particularly to further evaluate the extent to which euthymia may affect how a patient experiences the disease process [44].

## Conclusion

The findings of the present study indicated that the ES-J is a valid and highly sensitive clinimetric index, which can be used as a screening measure in the clinical process of assessment of recovery, particularly when symptoms are expected to be mild and/or when dealing with subclinical symptoms of psychological distress and depression. The findings of this study also support the use of the ES-J to detect vulnerability to depression and to identify subjects at higher risk of relapse.

## Data Availability

The datasets are available upon request from researchers with legitimate reasons.

## Abbreviations

DSM-5: The Diagnostic and statistical manual of mental disorders
ES: Euthymia scale
ES-J: The Japanese version of Euthymia scale
ISPOR: The International Society of Pharmacoeconomics and Outcomes Research
MDE: Major depressive episode
MINI: The Mini-International Neuropsychiatric Interview
WHO-5: The 5-item World Health Organization Well-Being Index
